# A Population-Based Registry Analysis on Hospitalized COVID-19 Patients with Previous Cardiovascular Disease: Clinical Profile, Treatment, and Predictors of Death

**DOI:** 10.3390/jcdd8120167

**Published:** 2021-11-29

**Authors:** Eduardo Gutiérrez-Abejón, Francisco Herrera-Gómez, Débora Martín-García, Eduardo Tamayo, Francisco Javier Álvarez

**Affiliations:** 1 Pharmacological Big Data Laboratory, Faculty of Medicine, University of Valladolid, 47005 Valladolid, Spain; fherrerag@saludcastillayleon.es (F.H.-G.); alvarez@med.uva.es (F.J.Á.); 2 Department of Pharmaceutical Assistance, Castilla y León Health Council, 47007 Valladolid, Spain; 3 Centro de Investigación Biomédica en Red de Enfermedades Infecciosas (Group CB21/13/00051), Carlos III Institute of Health, 28029 Madrid, Spain; 4 Transplantation Center, Lausanne University Hospital and University of Lausanne, CH-1011 Lausanne, Switzerland; 5 Department of Kidney Resuscitation and Acute Purification Therapies, Complejo Asistencial de Zamora, 49022 Zamora, Spain; 6 BioCritic, Group for Biomedical Research in Critical Care Medicine, Faculty of Medicine, University of Valladolid, 47005 Valladolid, Spain; tamayo@med.uva.es; 7 Department of Nephrology, Hospital Clínico Universitario de Valladolid, 47003 Valladolid, Spain; deboramarg@yahoo.es; 8 Department of Anesthesiology, Hospital Clínico Universitario de Valladolid, 47003 Valladolid, Spain; 9 Department of Surgery, Faculty of Medicine, University of Valladolid, 47005 Valladolid, Spain; 10 CEIm, Hospital Clínico Universitario de Valladolid, 47003 Valladolid, Spain

**Keywords:** SARS-CoV-2, COVID-19, cardiovascular diseases, heart failure, cerebrovascular diseases, clinical characteristics, treatment, mortality

## Abstract

A high percentage of patients with COVID-19 (coronavirus disease 2019) have previous cardiovascular disease (CVD). The findings presented here came from an epidemiological population-based registry study (real-world data) that enrolled all in-hospital COVID-19 patients with previous CVD from 1 March to 31 May 2020. Death, other comorbidities, hospital stay variables, ventilation type, and main clinical outcomes were evaluated. In Castile and Leon, 35.83% of the 7307 in-hospital COVID-19 patients who participated in this study had previous CVD, particularly arrhythmias (48.97%), cerebrovascular disease (25.02%), ischemic heart disease (22.8%), and chronic heart failure (20.82%). Of the patients, 21.36% were men and more than 90% were over 65 years of age, and the mortality rate achieved 32.93%. The most used medicines were antibiotics (91.41%), antimalarials (73.3%), steroids (46.64%), and antivirals (43.16%). The main predictors of death were age over 65 years (OR: 5), ventilation needs (OR: 2.81), treatment with anti-SIRS (systemic inflammatory response syndrome) medicines (OR: 1.97), antivirals (OR: 1.74) or steroids (OR: 1.68), SIRS (OR: 5.75), SARS (severe acute respiratory syndrome) (OR: 2.44), or AKI (acute kidney injury) (OR: 1.63) occurrence. Chronic heart failure and cerebrovascular disease were associated with a worse clinical course of COVID-19, especially in men older than 65 years with diabetes who developed SIRS, SARS, or AKI.

## 1. Introduction

Since the beginning of the COVID-19 (coronavirus disease 2019) pandemic in China in December 2019, a significant prevalence of previous cardiovascular diseases (CVDs) among SARS-CoV-2 (severe acute respiratory syndrome coronavirus 2) infected patients has been observed [1,2,3,4,5]. In addition, these patients with underlying CVD have shown a worse disease prognosis with an increasing mortality [1,6,7,8,9], especially in elderly people [3], and due to the presence of several factors such as low cardiovascular reserve, increased metabolic demand, procoagulant activity, and dysregulated immunity and inflammation due to the underlying CVD [10,11].

The respiratory symptoms produced by COVID-19 are more severe in patients with previous CVD, which has been related to higher secretion of angiotensin converting enzyme 2 (ACE2) in such patients compared to healthy subjects [3]. ACE2 is an aminopeptidase involved in heart function, which has been proposed as the entry site for SARS-CoV-2 [12], via receptor-mediated endocytosis [13]. Approximately 7.5% of myocardial cells show positive ACE2 activity, which may explain the virus’ entry into cardiomyocytes and the underlying cardiotoxicity [14]. According to some authors, ACE2 is related to a worse outcome in males with COVID-19 compared to females. First, the ACE2 gene is located on the X chromosome; thus, the expression of ACE2 will be higher in males than in females. Second, hypertension and heart failure are associated with an elevated serum ACE2 level in males [15]. Furthermore, ACE2 inhibition has been related to cytokine storm, which together with increased metabolic demand and procoagulant activity explains, to some extent, the worse clinical outcomes in COVID-19 patients with previous CVD [16,17,18].

Cytokine storm, characterized by the overproduction of pro-inflammatory cytokines and chemokines, such as tumor necrosis factor-α, IL-1β, and IL-6, can result in vascular inflammation, plaque instability, myocardial inflammation, hypercoagulability, and direct myocardial suppression [19,20,21] and, as a consequence, in serious cardiovascular damages such as tachycardia, hypotension, and left ventricular dysfunction [22]. In the same sense, cardiac injury can be influenced by other systemic consequences of COVID-19 infection, such as sepsis and disseminated intravascular coagulation (DIC) [23], characterized by increased d-dimer levels, which is observed in 71.4% of deceased patients [24].

One should be aware of medicine related heart damage during COVID-19 treatment [3], except for remdesevir [25], that are used in an off-label manner with relevant side effects and cardiovascular toxicity [26]. Antivirals can cause heart failure, arrhythmia, and other cardiovascular disorders [3], anti-inflammatory agents, such as hydrochloroquine and chloroquine, are associated with a cardiotoxicity risk, and corticosteroids, such as methylprednisolone, can cause water retention and consequent ionic imbalance [27].

This study was carried out in Castile and Leon, the largest region in Spain with a population of 2,323,770 inhabitants. The public healthcare system has 7141 beds spread over 14 hospitals, of which five are first-level referral hospitals, six are general hospitals, and three are regional hospitals. As in the rest of the world, at the beginning of the COVID-19 pandemic, the health system “collapsed”, and rapid therapeutic decisions had to be made based on poor existing scientific evidence. In this sense, each hospital developed its own pharmacological protocol based on national standards [28,29]. On the assumption that in patients with previous CVD, the clinical course of COVID-19 is associated with a worse prognosis, the main aim of this study was to provide real-world data on the influence of previous CVD on the hospital death of patients with COVID-19 as well as discerning what type of CVD has the most marked influence on a possible fatal outcome. In this way, the prevalence of in-hospital COVID-19 patients affected by previous CVD in Castile and Leon, taking gender into consideration, is described. In addition, the pattern of pharmacological treatment, mortality rate, and the main predictors of mortality were analyzed.

## 2. Materials and Methods

### 2.1. Real-World Study Details

The findings presented here came from an epidemiological population-based registry study (real-world data). The study was carried out following the main standards established for observational studies, the Reporting of Studies Conducted using Observational Routinely Collected Health Data (RECORD) [30] recommendations, and the Strengthening the Reporting of Observational Studies in Epidemiology (STROBE) [31] standards. This study was approved by the East Valladolid Area Ethics Committee on 11 June 2020 (reference: PI 20-1863).

The study period covered from 1 March 2020 to 31 May 2020, and all in-hospital COVID-19 patients with previous CVD were included. The Castile and Leon public hospital network includes 14 hospitals with a total capacity of 7141 beds to cover a population of 2,323,770 inhabitants. To facilitate analysis, biweekly periods were established.

Previous CVDs were established using the International Classification of Diseases, 10th revision (ICD-10). The following categories were considered: aneurysms, arrhythmias, atherosclerosis, heart dysfunction, cerebrovascular disease, ischemic heart disease, chronic heart failure, cardiomyopathy, and thrombosis–thrombophlebitis. In addition, other comorbidities, such as hypertension, diabetes, obesity (BMI ≥ 30 kg/m^2^), chronic kidney diseases, chronic respiratory disease, neoplasia, and autoimmune disease were considered.

Data were obtained from different healthcare databases: Jimena and Medora (https://www.saludcastillayleon.es/sanidad/cm, accessed on 6 May 2021), electronic medical records from hospitals and primary healthcare centers, respectively; Pestadístico (https://pestadistico.inteligenciadegestion.mscbs.es/publicoSNS/N/rae-cmbd/rae-cmbd, accessed on 6 May 2021), hospital discharges information in Castile and Leon; CONCYLIA pharmaceutical care information system (http://www.saludcastillayleon.es/portalmedicamento/es/indicadoresinformes/concylia, accessed on 6 May 2021), hospital medicine dispensing.

### 2.2. Variables

The main study outcome was in-hospital patient death. Hospital stay variables were considered such as hospitalization period (in days) and length of stay in the intensive care unit (ICU) (in days). Furthermore, ventilation type data were obtained: oxygenation, non-invasive positive pressure ventilation (NIPPV), and invasive ventilation (IV). Finally, the following clinical outcomes were evaluated: severe acute respiratory syndrome (SARS), acute kidney injury (AKI), systemic inflammatory response syndrome (SIRS), acute heart failure, bacterial and fungal superinfection, and DIC.

For medicine selections, the national recommendations [28,29] were followed and grouped according to the Anatomical Therapeutic Chemical Code (ATC): antibiotics, antimalarials, steroids, antivirals, tocilizumab, and other anti-SIRS medicines (Supplementary Materials Table S1).

### 2.3. Statistical Analysis

For all analyses, the gender and age distribution of the population were considered, establishing two groups with a cut-off age of 65 years. The findings related to the prevalence of previous CVD, comorbidities, pharmacological treatment, and clinical outcomes are expressed in frequencies (percentage) with their corresponding 95% confidence intervals (95% CI) and as means accompanied by their standard deviations (SDs) or as medians accompanied by their interquartile range (IQR).

For comparisons between groups, the Student’s *t*-test or the Mann–Whitney U test for continuous variables and Pearson’s chi-square test or Fisher’s exact test for categorical variables were used, as appropriate. The normal data distribution was evaluated using the Kolmogorov–Smirnov and Shapiro–Wilk tests.

To identify the predictors of in-hospital patients’ death with previous CVD, a multivariate logistic regression with a forward selection approach was performed. The results are expressed in terms of adjusted odds ratio (OR) and 95% CI, and the following variables were included in the model: age (≥65), gender, ventilation, type of previous CVD (aneurysms, arrhythmias, atherosclerosis, heart dysfunction, cerebrovascular disease, ischemic heart disease, chronic heart failure, cardiomyopathy, and thrombosis–thrombophlebitis), other comorbidities (hypertension, diabetes, chronic kidney diseases, obesity, chronic respiratory disease, neoplasia, and autoimmune disease), obesity, medication use (antibiotics, antimalarials, steroids, antivirals, tocilizumab, and anti-SIRS), and clinical outcomes (SARS, SIRS, DIC, acute heart failure, and bacterial and fungal superinfections).

The level of significance was set at *p* ≤ 0.05. All statistical analyses were performed using the Statistical Package for the Social Sciences (SPSS version 24.0., SPSS Inc., Chicago, IL, USA).

## 3. Results

### 3.1. Clinical Findings

In Castile and Leon, 7307 patients were hospitalized for COVID-19 between 1 March 2020 and 31 May 2020, 2618 (35.83%) of whom had previous CVD. Among patients with COVID-19 and previous CVD, more male than female (59.63% vs. 40.37%; *p* = 0.001) and approximately 90% of the patients were older than 65 years with a mortality rate of 32.93%. With respect to previous CVD, arrhythmias (48.97%), cerebrovascular disease (25.02%), ischemic heart disease (22.8%), and chronic heart failure (20.82%) were observed. Regarding the rest of the comorbidities, the following were representative: hypertension (66%), diabetes mellitus (29.56%), chronic respiratory disease (19.86%), neoplasia (14.21%), and chronic kidney disease (12.03%). SARS (15.51%), AKI (14.86%), and SIRS (4.05%) occurred among the participants (Table 1). The percentage of obesity was higher in patients with previous CVD (25.62% vs. 15.14%; *p* = 0.001), being higher in female than in male (27.82% vs. 24.08%; *p* = 0.001).

Medians for hospitalization and ICU length of stay were 10 (6–16) days and 14 (7–30) days, respectively. The most used ventilation mode was IV (3.13%), being almost four times higher in male than in female (4.61% vs. 0.95%; *p* = 0.001). On the other hand, the most used medicines were antibiotics (91.41%), antimalarials (73.3%), steroids (46.64%), and antivirals (43.16%) (Table 1).

**Figure 1:**
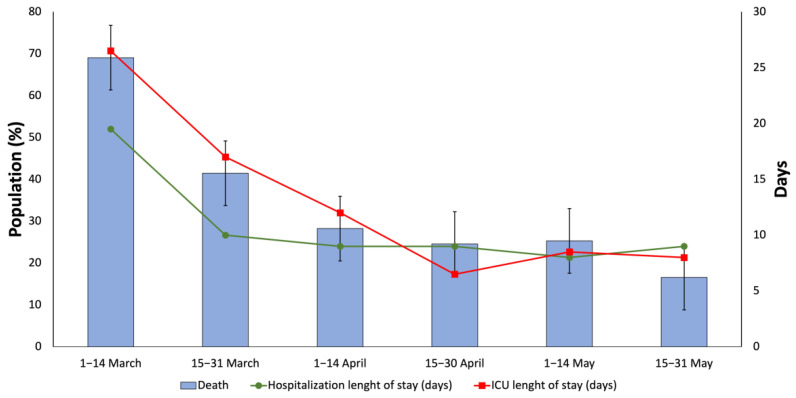
Biweekly clinical baselines in hospitalized COVID-19 patients with previous cardiovascular disease (1 March 2020–31 May 2020).

The mortality rate has decreased from 1 March 2020 and 15 March 2020, (69.05%) to 15 May 2020 and 31 May 2020 (16.55%). The hospital and ICU length of stay also decreased throughout the study period, from a median of 19.5 to 9 days and 26.5 to 8 days, respectively (Figure 1).

### 3.2. Pharmacological Treatment

Table 2 shows the most used medicines grouped by type: ceftriaxone (70.24%) and azithromycin (67.49%) (antibiotics), hydroxychloroquine (69.14%) (antimalarials), methylprednisolone (43.32%) (steroids), lopinavir–ritonavir (43.12%) (antivirals), and interferon beta (6.38%) (other anti-SIRS). Among non-deceased patients, antimalarials were used more frequently, specifically hydroxychloroquine (*p* = 0.005), while among deceased patients, steroids, such as methylprednisolone (*p* = 0.001), and other anti-SIRS (*p* = 0.001) were used more frequently (Table 2).

**Figure 2:**
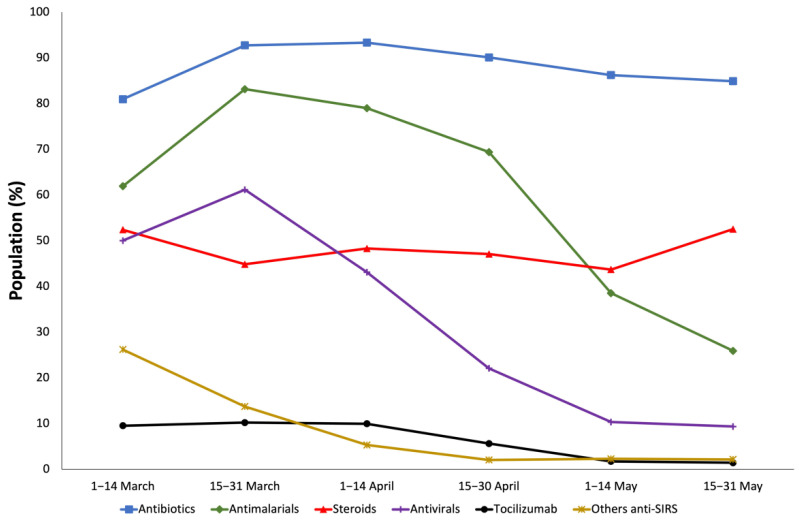
Therapy evolution in hospitalized COVID-19 patients with previous cardiovascular disease (1 March 2020–31 May 2020).

Between 1 March 2020 and 31 May 2020, the use of antibiotics and steroids remained stable. On the other hand, a decrease in the use of antimalarials (61.9–25.9%), antivirals (50–9.35%), tocilizumab (9.52–1.44%), and other anti-SIRS, especially interferon beta (26.19–1.44%) was observed (Supplementary Materials Table S2 and Figure 2).

### 3.3. Risk Factor for Clinical Outcomes and Medication Prescribed

The multiple logistic regression analysis for all in-hospital COVID-19 patients showed that previous CVD impact on death (OR: 1.57; 95% CI: 1.39–1.79). Furthermore, in patients with previous CVD, the presence of comorbidities, such as diabetes mellitus, was associated with a higher probability of death compared to those patients with previous CVD without comorbidities (OR: 1.27, 1.05–1.55). In patients with previous CVD, it was observed that patients with chronic heart failure (OR: 1.27, 1.02–1.58) and with cerebrovascular disease (OR: 1.35, 1.09–1.66) were most likely to die than those who had other types of previous CVD.

In addition, among in-hospital COVID-19 patients, death was more likely to occur in those over 65 years of age (OR: 5, 3.32–7.52), in males (OR: 1.36, 1.12–1.65), in those needing ventilation (OR: 2.81, 1.97–4), in those treated with anti-SIRS drugs (OR: 1.97, 1.39–2.81), antivirals (OR: 1.74, 1.39–2.17), and steroids (OR: 1.68, 1.39–2.02), and in those who developed SIRS (OR: 5.75, 2.53–13.07), SARS (OR: 2.44, 1.91–3.12), or AKI (OR: 1.63, 1.27–2.11).

## 4. Discussion

Approximately one out of three in-hospital COVID-19 patients had previous CVD, mainly arrhythmias, cerebrovascular disease, ischemic heart disease, and chronic heart failure. The mortality rate of these patients was the third highest, after those who developed AKI (46.1%) and SARS (42.53%). As in other studies conducted by our team [32,33], the use of antibiotics and steroids remained stable, while the use of antimalarials, antivirals, tocilizumab, and other anti-SIRS decreased between 1 March 2020 and 31 May 2020. Our results show that males with previous CVD, especially chronic heart failure or cerebrovascular diseases, older than 65 years, who needed some type of ventilation, and who developed SIRS, SARS, and AKI had a higher risk of death. In addition, the use of anti-SIRS medicines, antivirals, and steroids was related to a worse prognosis of the disease.

At this point, it is convenient to clarify the dual role of ACE2. First, COVID-19 symptoms were more severe in patients with CVD, which might be associated with increased secretion of ACE2 in these patients compared with healthy individuals [3]. Second, the inhibition of ACE2 may be another factor of lung injury as well as the cause of the systemic inflammation following cytokine release, which can result in acute respiratory distress syndrome (ARDS) and multiorgan dysfunction [16,17,18].

The prevalence of in-hospital COVID-19 patients with previous CVD is higher than other Spanish [34,35], European [36], and Chinese [1] cohorts, while it is lower compared to another US cohort [37]. This finding is possible in relation to the predominance of the elderly in Castile and Leon, which is demonstrated by the elevated median age observed (83 years). As in other national [34,35] and international cohorts [38,39,40,41], our findings showed a higher prevalence in males than in females. A meta-analysis carried out by Ortolan et al. [42] showed that males are 61% more likely to die from COVID-19 infection than females. This higher predisposition in males appears to be related to differences in innate immunity, steroid hormones, and factors related to sex chromosomes. In this sense, the double copy of the X chromosome in females is essential against viral infections. However, it is observed that female are at a higher risk of COVID-19 clinical manifestations in the long term than males [43].

Surprisingly, in our cohort, hospital death was not influenced by the obesity factor as was the case in other studies [44,45]. One possible explanation is that the obesity rate of our patients was lower than in other cohorts [46].

Although primarily a respiratory disease, COVID-19 also causes important systemic effects, especially on the cardiovascular and immune systems. For this reason, in patients with previous CVD, mortality rates of 5–10 times higher are observed [8]. Our findings showed that the increase in mortality was not so pronounced among patients without previous CVD (19.68%) compared to patients with previous CVD (32.93%).

As in other published studies [38,40,41,47,48,49], hypertension and diabetes were the most observed comorbidities, followed by chronic respiratory disease and some type of neoplasia, increasing the thrombotic event risk, which is associated with a mortality increase [50].

The pharmacological treatments used for COVID-19 have significant cardiovascular toxicities and side effects. However, data on side effects come from chronic treatments for other clinical conditions such as autoimmune diseases (hydroxychloroquine, chloroquine, and tocilizumab), hepatitis (interferon), or HIV (lopinavir–ritonavir) [26].

Azithromycin was consumed mainly by patients who did not die (69.36%), although it is known that this antibiotic in combination with hydroxychloroquine prolongs the QT interval [51].

Antimalarial drugs (i.e., hydroxychloroquine and chloroquine) were used in three out of four patients. It is known that these drugs at high doses mainly cause cardiac conduction disorders, in addition to ventricular hypertrophy, hypokinesia, heart failure, pulmonary arterial hypertension, and valve dysfunction [52].

Steroids were used in half of deceased patients (51.51%) and a positive OR was observed (1.68). This may be since in the case of methylprednisolone, which was the most frequently used steroid, it has been associated with electrolyte derangements, fluid retention, and hypertension [53].

The percentage of patients treated with antivirals (43.16%) was lower than in other studies [54], but it has been related to a higher probability of death (OR: 1.74). The combination of lopinavir–ritonavir must be used with caution, since it can interact with cardiovascular medicines metabolized by cytochrome P450-3A4 such as antiarrhythmics, antiplatelets, and anticoagulants [55], in addition to causing QT and PR segment prolongation [56]. In the case of remdesevir, no associated cardiovascular adverse effects have been reported [25].

The use of tocilizumab (8.33%) has demonstrated cardiovascular safety in patients with rheumatoid arthritis, although it is not currently confirmed in patients with previous CVD [57].

The anti-SIRS medicines are associated with a greater probability of death (OR: 1.97), probably because the patients are in a more advanced and more severe phase of the disease; therefore, the probability of death is higher.

Regarding thromboprophylaxis strategies, according to different studies [58,59], initial treatment with heparin improves the prognosis of COVID-19 by increasing survival until hospital discharge compared to routine thromboprophylaxis, especially in non-critical patients.

Lastly, this study has several limitations that must be mentioned. Occasionally, the diagnosis of COVID-19 was made according to clinical and radiological criteria without microbiological confirmation. Another possible limitation was the unavailability of other variables, such as earlier lifestyle factors, physical activity level, or sleep, which can influence the results of the logistic regression model regarding the prediction of hospital death in patients with previous CVD. Regarding pharmacological treatment, only the Spanish guidelines [28,29] indicators were considered, and other possible treatments used may have been ignored. On the other hand, cardiovascular pharmacological treatments during patient admission were not available, which may be a limitation.

## 5. Conclusions

Almost one out of three in-hospital COVID-19 patients had a previous CVD, of which half had arrhythmias history. In addition, these patients had a worse disease prognosis with higher mortality than the global patient average [32].

According to our findings, men older than 65 years with diabetes who developed SIRS, SARS, or AKI had a higher probability of hospital death. Among previous CVDs, chronic heart failure and cerebrovascular disease were associated with a worse clinical course of the disease, and a greater probability of associated death was observed.

Regarding pharmacological treatment, antivirals and antimalarials, especially, have shown long-term cardiovascular toxicity, but it is too early to analyze their short-term effects. The medicines used for COVID-19 treatment were modified throughout the study period according to the availability and the national guideline recommendations [28,29].

Definitely, previous CVDs are related to a worse prognosis of COVID-19; therefore, “special attention” to these patients must be paid, especially after steroid, antiviral, and anti-SIRS administration, which are associated with a higher probability of death.

## Figures and Tables

**Table 1:** Baseline characteristics and clinical outcomes of hospitalized COVID-19 patients with previous cardiovascular disease (1 March 2020–31 May 2020).

		TOTAL	MALE	FEMALE	*p*
**N**		2618	1561	1057	0.001
Age (median and IQR)		83 (73–88)	80 (71–86)	85 (76–90)	0.001
Age < 65 (95% CI)		12.18 (10.93–13.44)	13 (11.34–14.67)	10.97 (9.09–12.86)	0.013
Age >= 65 (95% CI)		87.82 (86.56–89.07)	87 (85.33–88.66)	89.03 (87.14–90.91)	0.013
**Chronic diseases (95% CI)**					
Hypertension		66 (64.19–67.82)	62.97 (60.58–65.37)	70.48 (67.73–73.23)	0.001
Diabetes		29.56 (27.82–31.31)	30.62 (28.33–32.91)	28 (25.3–30.71)	0.162
Chronic respiratory diseases		19.86 (18.33–21.39)	22.87 (20.79–24.95)	15.42 (13.24–17.6)	0.001
Neoplasia		14.21 (12.87–15.55)	16.27 (14.44–18.1)	11.16 (9.27–13.06)	0.001
Chronic kidney disease		12.03 (10.79–13.28)	12.11 (10.49–13.73)	11.92 (9.97–13.87)	0.903
Autoimmune disease		8.21 (7.16–9.26)	8.58 (7.19–9.97)	7.66 (6.06–9.27)	0.425
**Previous cardiovascular disease (95% CI)**					
Arrhythmias		48.97 (47.05–50.88)	48.11 (45.63–50.59)	50.24 (47.22–53.25)	0.286
Cerebrovascular disease		25.02 (23.36–26.68)	23.77 (21.66–25.88)	26.87 (24.2–29.54)	0.072
Ischemic heart disease		22.8 (21.2–24.41)	27.48 (25.27–29.7)	15.89 (13.69–18.1)	0.001
Chronic heart failure		20.82 (19.26–22.37)	17.55 (15.67–19.44)	25.64 (23.01–28.27)	0.001
Thrombosis–thrombophlebitis		16.73 (15.3–18.16)	14.29 (12.55–16.02)	20.34 (17.91–22.77)	0.001
Atherosclerosis		10.73 (9.55–11.92)	12.3 (10.67–13.93)	8.42 (6.75–10.09)	0.02
Aneurysms		8.79 (7.7–9.87)	12.36 (10.73–14)	3.5 (2.39–4.61)	0.001
Cardiomyopathy		1.99 (1.45–2.52)	2.63 (1.83–3.42)	1.04 (0.43–1.65)	0.004
Heart dysfunction		1.3 (0.87–1.73)	1.22 (0.67–1.76)	1.42 (0.71–2.13)	0.654
**Treatment**					
Oxygen delivery and ventilation (95% CI)					
IV		3.13 (2.46–3.8)	4.61 (3.57–5.65)	0.95 (0.36–1.53)	0.001
Oxygen delivery		2.94 (2.29–3.59)	3.33 (2.44–4.22)	2.37 (1.45–3.28)	0.159
NIPPV		2.06 (1.52–2.61)	2.63 (1.83–3.42)	1.23 (0.57–1.89)	0.014
*Medicines* (95% CI)					
Antibiotics		91.41 (90.33–92.48)	91.1 (89.68–92.51)	91.86 (90.22–93.51)	0.491
Antimalarials		73.3 (71.61–74.99)	75.98 (73.86–78.1)	69.35 (66.57–72.13)	0.001
Steroids		46.64 (44.73–48.55)	49.84 (47.36–52.32)	41.91 (38.94–44.89)	0.001
Antivirals		43.16 (41.27–45.06)	47.28 (44.8–49.75)	37.09 (34.17–40)	0.001
Tocilizumab		8.33 (7.27–9.39)	11.08 (9.53–12.64)	4.26 (3.04–5.47)	0.001
Other anti-SIRS *		8.21 (7.16–9.26)	10.95 (9.41–12.5)	4.16 (2.96–5.37)	0.001
**Clinical Outcomes**					
Hospital LoS (median and IQR)		10 (6–16)	10 (6–17)	9 (6–16)	0.111
ICU LoS (median and IQR)		14 (7–30)	14 (7–29)	13.5 (6–30)	0.969
N (Number of patients admitted to the ICU)		159	133	26	
Death (95% CI)		32.93 (31.13–34.73)	35.81 (33.43–38.19)	28.67 (25.94–31.39)	0.001
SARS (95% CI)		15.51 (14.12–16.89)	18 (16.1–19.91)	11.83 (9.88–13.77)	0.001
AKI (95% CI)		14.86 (13.5–16.22)	15.44 (13.65–17.23)	14 (11.91–16.09)	0.311
SIRS (95% CI)		4.05 (3.29–4.8)	4.23 (3.23–5.23)	3.78 (2.63–4.93)	0.543
Bacterial superinfection (95% CI)		3.4 (2.71–4.09)	3.72 (2.78–4.65)	2.93 (1.92–3.95)	0.278
Fungal superinfection (95% CI)		2.29 (1.72–2.87)	2.05 (1.35–2.75)	2.65 (1.68–3.62)	0.315
Acute heart failure (95% CI)		2.1 (1.55–2.65)	2.88 (2.05–3.71)	0.95 (0.36–1.53)	0.001
DIC (95% CI)		0.19 (0.02–0.36)	0.32 (0.04–0.6)	0 (0–0)	0.066

* Anakinra, baricitinib, interferon, ruxolitinib, and siltuximab. 95% CI, confidence interval; IQR, interquartile range; IV, invasive ventilation; NIPPV, non-invasive positive pressure ventilation; SIRS, systemic inflammatory response syndrome; ICU, intensive care unit; LoS, length of stay; SARS, severe acute respiratory syndrome; AKI, acute kidney injury; DIC, disseminated intravascular coagulation.

**Table 2:** Pharmacological treatment in hospitalized COVID-19 patients with previous cardiovascular disease (1 March 2020–31 May 2020).

Medicines	TOTAL (95% CI)	No Death (95% CI)	Death (95% CI)	*p*
	*N* = 2618	*N* = 1756	*N* = 862	
**Antibiotics**	**91.41 (90.33–92.48)**	**91.69 (90.32–93.06)**	**90.84 (89.1–92.57)**	**0.466**
Ceftriaxone	70.24 (68.49–72)	69.93 (67.66–72.21)	70.88 (68.14–73.62)	0.617
Azithromycin	67.49 (65.7–69.29)	69.36 (67.08–71.65)	63.69 (60.79–66.59)	0.004
Levofloxacin	16.31 (14.89–17.73)	14.69 (12.94–16.45)	19.61 (17.21–22)	0.001
Cefditoren	2.52 (1.92–3.12)	3.25 (2.37–4.13)	1.04 (0.43–1.66)	0.001
Teicoplanin	1.57 (1.09–2.04)	1.14 (0.61–1.67)	2.44 (1.51–3.37)	0.012
Clarithromycin	0.42 (0.17–0.67)	0.4 (0.09–0.71)	0.46 (0.05–0.87)	0.808
Moxifloxacin	0.19 (0.02–0.36)	0.11 (0.02–0.33)	0.35 (0.01–0.72)	0.197
Cefotaxime	0.19 (0.02–0.36)	0.23 (0.01–0.48)	0.12 (0.01–0.23)	0.538
Ceftaroline	0.11 (0.01–0.27)	0.17 (0.03–0.44)	0 (0–0)	0.225
**Antimalarials**	**73.3 (71.61–74.99)**	**74.54 (72.38–76.71)**	**70.77 (68.02–73.51)**	**0.041**
Hydroxychloroquine	69.14 (67.37–70.91)	70.9 (68.65–73.15)	65.55 (62.68–68.41)	0.005
Chloroquine	5.5 (4.63–6.37)	4.78 (3.72–5.84)	6.96 (5.43–8.49)	0.022
**Steroids**	**46.64 (44.73–48.55)**	**44.25 (41.78–46.71)**	**51.51 (48.5–54.52)**	**0.001**
Methylprednisolone	43.32 (41.42–45.21)	40.26 (37.83–42.69)	49.54 (46.52–52.55)	0.001
Prednisone	10.66 (9.48–11.84)	12.41 (10.78–14.05)	7.08 (5.53–8.62)	0.001
**Antivirals**	**43.16 (41.27–45.06)**	**42.26 (39.8–44.71)**	**45.01 (42.01–48.01)**	**0.181**
Lopinavir-Ritonavir	43.12 (41.23–45.02)	42.2 (39.75–44.65)	45.01 (42.01–48.01)	0.172
Remdesevir	0.08 (0.01–0.22)	0.11 (0.02–0.33)	0 (0–0)	0.322
**Tocilizumab**	**8.33 (7.27–9.39)**	**8.83 (7.42–10.23)**	**7.31 (5.74–8.88)**	**0.186**
**Others anti SIRS**	**8.21 (7.16–9.26)**	**7 (5.74–8.27)**	**10.67 (8.81–12.53)**	**0.001**
Interferon Beta	6.38 (5.44–7.32)	5.3 (4.19–6.41)	8.58 (6.9–10.27)	0.001
Anakinra	1.45 (0.99–1.91)	1.37 (0.79–1.94)	1.62 (0.86–2.39)	0.605
Baricitinib	0.38 (0.15–0.62)	0.46 (0.12–0.79)	0.23 (0.02–0.55)	0.384
Ruxolitinib	0.11 (0.01–0.27)	0.11 (0.02–0.33)	0.12 (0.01–0.23)	0.988
Siltuximab	0.08 (0.01–0.22)	0 (0–0)	0.23 (0.02–0.55)	0.043

95% CI, confidence interval; SIRS, systemic inflammatory response syndrome.

## Data Availability

Restrictions apply to the availability of these data. Data were obtained from regional health authorities (Gerencia Regional de Salud (GRS)) and may be requested from sdinvestigacion@saludcastillayleon.es (GRS).

## Supplementary Materials

The following are available online at https://www.mdpi.com/article/10.3390/jcdd8120167/s1, Table S1: List of medicines used in the COVID-19 treatment according to Spanish guidelines; Table S2: Treatment and clinical outcome evolution of in-hospital COVID-19 patients with previous cardiovascular diseases.
