# Supplementary material for: A Population-Based Registry Analysis on Hospitalized COVID-19 Patients with Previous Cardiovascular Disease: Clinical Profile, Treatment, and Predictors of Death
Source: J Cardiovasc Dev Dis. 2021 Nov 29;8(12):167. doi: 10.3390/jcdd8120167 (PMC8705942; doi:10.3390/jcdd8120167)
Supplement: Supplementary file 1 [file jcdd-08-00167-s001.zip › jcdd-1431127-supplementary.pdf]

Supplementary Table S1. List of medicines used in the COVID-19 treatment according to Spanish guidelines [26,27].

Medicines Type	ATC Code	Medicine	Medicines Type	ATC Code	Medicine
Antibiotics	J01DD01	Cefotaxime	Anti SIRS Drugs	L01XE18	Ruxolitinib
	J01DD04	Ceftriaxone		L03AB05	Interferon alpha 2b
	J01DD16	Cefditoren		L03AB08	Interferon beta 1b
	J01DI02	Ceftaroline		L04AA37	Baricitinib
	J01FA09	Clarithromycin		L04AC03	Anakinra
	J01FA10	Azithromycin		L04AC07	Tocilizumab
	J01MA12	Levofloxacin		L04AC11	Siltuximab
	J01MA14	Moxifloxacin		L04AC14	Sarilumab
	J01XA02	Teicoplanine	Antivirals	J05AR10	Lopinavir and Ritonavir
Antimalarials	P01BA01	Chloroquine		J05AX95*	Remdesivir
	P01BA02	Hidroxychloroquine	Steroids	H02AB04	Methylprednisolone
				H02AB07	Prednisone

Abbreviations: SIRS, systemic inflammatory response syndrome.

Supplementary Table S2. Treatment and clinical outcomes evolution of in-hospital COVID-19 patients with previous cardiovascular diseases (March 1st - May 31st, 2020).

**Medicines**	**1-14 March**	**15-31 March**	**1-14 April**	**15-30 April**	**1-14 May**	**15-31 May**
	N=42	N=1086	N=733	N=444	N=174	N=139
**Antibiotics**	**80.95 (69.08-92.83)**	**92.73 (91.18-94.27)**	**93.32 (91.83-94.8)**	**90.09 (88.31-91.87)**	**86.21 (84.16-88.26)**	**84.89 (82.76-87.02)**
Ceftriaxone	52.38 (37.28-67.49)	73.66 (71.05-76.28)	70.53 (67.82-73.24)	66.67 (63.86-69.47)	67.82 (65.04-70.59)	61.87 (58.98-64.76)
Azithromycin	33.33 (19.08-47.59)	70.81 (68.11-73.51)	73.26 (70.63-75.89)	65.77 (62.94-68.59)	51.72 (48.75-54.7)	46.76 (43.8-49.73)
Levofloxacin	38.1 (23.41-52.78)	18.05 (15.76-20.34)	11.73 (9.82-13.65)	16.67 (14.45-18.88)	17.24 (14.99-19.49)	17.99 (15.7-20.27)
Clarithromycin	2.38 (0.23-4.99)	0.18 (0.07-0.30)	0 (0-0)	0.9 (0.34-1.46)	0.57 (0.13-1.02)	2.16 (1.29-3.02)
Teicoplanin	0 (0-0)	1.47 (0.76-2.19)	2.18 (1.31-3.05)	1.13 (0.5-1.75)	1.15 (0.52-1.78)	1.44 (0.73-2.15)
Cefditoren	0 (0-0)	2.39 (1.48-3.3)	2.59 (1.65-3.54)	2.93 (1.93-3.93)	2.3 (1.41-3.19)	2.88 (1.88-3.87)
Cefotaxime	0 (0-0)	0.09 (0.01-0.18)	0.27 (0.04-0.50)	0.23 (0.06-0.39)	0.57 (0.13-1.02)	0 (0-0)
Moxifloxacin	0 (0-0)	0 (0-0)	0.55 (0.11-0.98)	0.23 (0.06-0.39)	0 (0-0)	0 (0-0)
Ceftaroline	0 (0-0)	0.18 (0.07-0.30)	0 (0-0)	0.23 (0.06-0.39)	0 (0-0)	0 (0-0)
**Antimalarials**	**61.9 (47.22-76.59)**	**83.15 (80.92-85.38)**	**78.99 (76.57-81.41)**	**69.37 (66.63-72.11)**	**38.51 (35.61-41.4)**	**25.9 (23.29-28.5)**
Hydroxycloroquine	59.52 (44.68-74.37)	76.7 (74.19-79.22)	74.76 (72.18-77.34)	68.69 (65.94-71.45)	37.36 (34.48-40.23)	24.46 (21.9-27.02)
Cloroquine	4.76 (0.68-9.2)	9.48 (7.74-11.23)	4.23 (3.03-5.43)	0.68 (0.19-1.16)	1.15 (0.52-1.78)	2.16 (1.29-3.02)
**Steroids**	**52.38 (37.28-67.49)**	**44.84 (41.89-47.8)**	**48.29 (45.32-51.27)**	**47.07 (44.1-50.04)**	**43.68 (40.73-46.63)**	**52.52 (49.55-55.49)**
Methylprednisolone	47.62 (32.51-62.72)	42.08 (39.14-45.02)	44.61 (41.65-47.57)	45.05 (42.09-48)	39.66 (36.75-42.56)	43.88 (40.93-46.84)
Prednisone	14.29 (3.7-24.87)	9.02 (7.32-10.73)	12.01 (10.07-13.94)	10.81 (8.96-12.66)	12.07 (10.13-14.01)	12.95 (10.95-14.95)
**Antivirals**	**50 (34.88-65.12)**	**61.14 (58.24-64.04)**	**43.11 (40.17-46.06)**	**22.07 (19.61-24.54)**	**10.34 (8.53-12.16)**	**9.35 (7.62-11.08)**
Lopinavir-Ritonavir	50 (34.88-65.12)	61.05 (58.15-63.95)	43.11 (40.17-46.06)	22.07 (19.61-24.54)	10.34 (8.53-12.16)	9.35 (7.62-11.08)
Remdesevir	0 (0-0)	0.18 (0.07-0.30)	0 (0-0)	0 (0-0)	0 (0-0)	0 (0-0)
**Tocilizumab**	**9.52 (0.65-18.4)**	**10.22 (8.42-12.02)**	**9.96 (8.18-11.74)**	**5.63 (4.26-7)**	**1.72 (0.95-2.5)**	**1.44 (0.73-2.15)**
**Others anti-SIRS**	**26.19 (12.89-39.49)**	**13.72 (11.67-15.77)**	**5.32 (3.99-6.66)**	**2.03 (1.19-2.87)**	**2.3 (1.41-3.19)**	**2.16 (1.29-3.02)**
Interferon Beta	26.19 (12.89-39.49)	12.52 (10.55-14.49)	2.32 (1.42-3.21)	0 (0-0)	0 (0-0)	1.44 (1.04-2.84)
Anakinra	0 (0-0)	0.83 (0.29-1.37)	2.59 (1.65-3.54)	1.8 (1.01-2.59)	0.57 (0.13-1.02)	0.72 (0.22-1.22)
Baricitinib	0 (0-0)	0.09 (0.01-0.18)	0.68 (0.19-1.17)	0.23 (0.06-0.39)	1.72 (0.95-2.5)	0 (0-0)
Siltuximab	0 (0-0)	0.18 (0.07-0.30)	0 (0-0)	0 (0-0)	0 (0-0)	0 (0-0)
Ruxolitinib	0 (0-0)	0.18 (0.07-0.30)	0.14 (0.08-0.20)	0 (0-0)	0 (0-0)	0 (0-0)
Hospital LoS (median + IQR)	19.5 (9 -31)	10 (6-17)	9 (6-16)	9 (6-15)	8 (6-15)	9 (6-14)
ICU LoS (median + IQR)	26.5 (12-41)	17 (7-32)	12 (4-21)	6.5 (1-14)	8.5 (6-10)	8 (3-12)
	N=8	N=106	N=29	N=8	N=4	N=4
Death (% 95 CI)	69.05 (55.07-83.03)	41.44 (38.51-44.37)	28.24 (25.56-30.92)	24.55 (21.99-27.11)	25.29 (22.7-27.87)	16.55 (14.34-18.76)
SARS (% 95 CI)	19.05 (7.17-30.92)	19.43 (17.08-21.78)	14.87 (12.75-16.99)	11.49 (9.59-13.38)	10.34 (8.53-12.16)	6.47 (5.01-7.94)
AKI (% 95 CI)	7.14 (0.65-13.93)	12.15 (10.21-14.1)	16.51 (14.3-18.72)	18.24 (15.95-20.54)	22.41 (19.93-24.89)	9.35 (7.62-11.08)
SIRS (% 95 CI)	4.76 (0.68-9.2)	2.3 (1.41-3.19)	1.77 (0.99-2.56)	2.03 (1.19-2.87)	3.45 (2.36-4.53)	0 (0-0)
Bacterial superinfection (% 95	4.76 (0.68-9.2)	2.21 (1.34-3.08)	5.05 (3.75-6.35)	4.73 (3.47-5.99)	6.9 (5.39-8.4)	7.19 (5.66-8.73)
Fungal superinfection (% 95	2.38 (0.23-4.99)	2.03 (1.19-2.86)	3.68 (2.56-4.8)	3.83 (2.69-4.97)	9.77 (8-11.54)	3.6 (2.49-4.7)
Carodiomyopathy (% 95 CI)	4.76 (0.68-9.2)	2.12 (1.26-2.97)	2.18 (1.31-3.05)	3.38 (2.3-4.45)	1.72 (0.95-2.5)	0.72 (0.22-1.22)
DIC (% 95 CI)	2.38 (0.23-4.99)	0.18 (0.07-0.30)	0.14 (0.08-0.20)	0 (0-0)	0.57 (0.13-1.02)	0 (0-0)

Abbreviations: 95 CI, confidence interval, SARS, severe acute respiratory syndrome, AKI, acute kidney injury, SIRS, systemic inflammatory response syndrome, ICU, intensive care unit, LoS. length of stay, DIC, disseminated intravascular coagulation.
